# Effects of F/G-actin ratio and actin turn-over rate on NADPH oxidase activity in microglia

**DOI:** 10.1186/1471-2172-11-44

**Published:** 2010-09-08

**Authors:** Izabela Rasmussen, Line H Pedersen, Luise Byg, Kazuhiro Suzuki, Hideki Sumimoto, Frederik Vilhardt

**Affiliations:** 1 Dept. of Cellular and Molecular Medicine, The Panum Institute, Copenhagen University, 2200N Copenhagen, Denmark; 2 The Division of Biosignaling, National Institute of Health Sciences, 18-1 Kamiyoga 1-Chome, Tokyo, Japan; 3 Department of Biochemistry, Kyushu University Graduate School of Medical Sciences, Fukuoka 812-8582, Japan

## Abstract

**Background:**

Most *in vivo *studies that have addressed the role of actin dynamics in NADPH oxidase function in phagocytes have used toxins to modulate the polymerization state of actin and mostly effects on actin has been evaluated by end point measurements of filamentous actin, which says little about actin dynamics, and without consideration for the subcellular distribution of the perturbed actin cytoskeleton.

**Results:**

Here, we in addition to toxins use conditional expression of the major actin regulatory protein LIM kinase-1 (LIMK1), and shRNA knock-down of cofilin to modulate the cellular F/G-actin ratio in the Ra2 microglia cell line, and we use Fluorescence Recovery after Photobleaching (FRAP) in β-actin-YFP-transduced cells to obtain a dynamic measure of actin recovery rates (actin turn-over rates) in different F/G-actin states of the actin cytoskeleton. Our data demonstrate that stimulated NADPH oxidase function was severely impaired only at extreme actin recovery rates and F/G-actin ratios, and surprisingly, that any moderate changes of these parameters of the actin cytoskeleton invariably resulted in an increased NADPH oxidase activity.

**Conclusion:**

moderate actin polymerization and depolymerization *both *increase the FMLP and PMA-stimulated NADPH oxidase activity of microglia, which is directly correlated with neither actin recovery rate nor F/G- actin ratio. Our results indicate that NADPH oxidase functions in an enhanced state of activity in stimulated phagocytes despite widely different states of the actin cytoskeleton.

## Background

The superoxide-producing NADPH oxidase is expressed at high levels in professional phagocyte cells. It is composed of membrane subunits gp91phox (NOX-2) and p22phox, which form a heterodimeric flavocytochrome b_558 _complex (cyt b_558_), and cytosolic subunits p40phox, p47phox, p67phox, and the small GTPase Rac1 or 2 [1]. In resting cells p40phox, p47phox, and p67phox subunits exist in a cytosolic complex separated from cyt b_558_, but when NADPH oxidase is assembled and active, electrons abstracted from NADPH are channeled through gp91phox in the membrane to reduce molecular oxygen to superoxide radical on the extracellular aspect of the membrane. Translocation of cytosolic subunits to cyt b_558 _in the membrane occurs only following phagocyte activation by innate immune cell stimuli, which initiate intracellular signaling pathways leading to activation of Rac1 by GDP/GTP exchange factors, and phosphorylation of critical serine residues in p40phox and p47phox by serine/threonine kinases including PKCδ, AKT, and PAK1 (see [2] for references). This phosphorylation unmasks latent binding sites in p47phox for cyt b_558 _and also exposes the PHOX domains of p40phox and p47phox, which bind to phosphoinositol lipids in the membrane [3-5]. The role of p47phox [6], and for some stimuli also p40phox [7,8], seems therefore to be membrane targeting of p67phox, which together with Rac1/2 regulates electron transport of cyt b_558. _Translocation of Rac1 and the cytosolic phox proteins complex takes place simultaneously but independently of each other [2,9], and continuous exchange of cytosolic phox subunits and Rac1/2 are necessary to sustain production of superoxide during the respiratory burst [10,11].

The case for the actin cytoskeleton as an active participant in NADPH oxidase assembly and activity is supported by many observations in the literature including i) the acquisition of detergent-insolubility of cytosolic phox proteins in stimulated phagocytes, indicative of cytoskeleton association, ii) the co-localization and sometimes co-migratory (cytosol to membrane or vice versa) behavior of NADPH oxidase subunits with actin or actin-regulatory proteins in different cell types [12-15], and finally iii) the direct binding interactions between p40phox and p47phox with the actin regulatory proteins moesin [16], coronin [17], WAVE1 [15], Hic5/TRAF4 [13], and possibly cortactin [18,19], and the low affinity binding of p47phox to actin itself [20]. Additionally, cyt b_558 _is tightly associated with actin and is known to co-purify with actin and actin-associated proteins [21]. For these reasons it is widely believed that the actin cytoskeleton plays an active role in NADPH oxidase assembly and activation.

In the present study we demonstrate that F-actin polymerization and depolymerization can *both *increase NADPH oxidase activity in microglia, and that neither F/G-actin ratios nor actin turn-over rates are useful predictors of the effect of imposed actin rearrangement on NADPH oxidase activity.

## Methods

The murine microglia cell line Ra2 (licensed by the Japan Science and Technology Agency, Patent ID US6.673,6,5; JP3410738; EP10/602,234) was maintained in MEM with 10% FCS, 1 ng/ml GM-CSF (Peprotech, UK), and 5 μg/ml bovine insulin [22]. FMLP, PMA, luminol, latrunculin A, and HRP-II were purchased from Sigma (St. Louis, MO, USA) and jasplakinolide from Calbiochem (Darmstadt, Germany). Rabbit anti-LIMK1 antibodies were from Transduction Laboratories, and rabbit polyclonal anti-ser3(P)-cofilin antibodies from Cell Signaling Technology (Danvers, MA, USA). Alexa-conjugated phalloidin and secondary antibodies for immunofluorescence were all from Molecular Probes (Carlsbad, CA, USA).

### Lentivector construction

The human cDNA's coding for amino acids 4-647 of wild type LIMK1 (LIMK1-WT) and kinase dead LIMK1-D406A (LIMK1-DN), and β-actin-YFP (Clontech #6902-1; Mountain View, CA., USA) were inserted into the tetracycline-responsive lentiviral vector pLOX TW [23], and used to superinfect Ra2 045 cells expressing the tetracycline-responsive transactivator protein. For shRNA knock-down of mouse cofilin two DNA sequences containing the target sequences GGAGGACCTGGTGTTCATC (cofilin shRNA 1) and GGTGTTCAATGACATGAAG (cofilin shRNA 2) with loop sequence ACTCGAGA were synthesized, annealed, and cloned MluI/ClaI into lentivector pLVTHM-DsRed [24]. The vector was then used for superinfection of Ra2 tTR-KRAB cells expressing a tetracycline-responsive KRAB repressor protein, which in the absence of doxycycline represses transcription from polymerase I, II and III [24]. Expression of cofilin shRNA in Ra2 cells was induced 4-5 days at 50 ng/ml doxycycline before cells were used for experiments.

### Measurement of Superoxide production

FMLP or PMA-induced superoxide release was measured by luminol-enhanced chemiluminescence (luminol E-CL). Ra2 cells in HBSS buffer with 62.5 μM luminol and 2 U/ml HRP II were warmed two minutes in a 37°C water bath before distribution into Wallac isoplate wells (100.000 cells/well). Subsequently, superoxide production was measured as chemiluminescence before and after stimulation with 4 μM FMLP or 100 ng/ml PMA, respectively, delivered through the injector module of a thermostated Synergy HT microplate reader. With the luminol E-CL method used here, ca. 20-25% of the luminal E-CL signal probably derives from the formation of peroxynitrite via reaction of superoxide with NO, as discussed further in [25].

### Immunofluorescence and FRAP studies

For immunofluorescence Ra2 cells were fixed with 2% paraformaldehyde in phosphate-buffer pH 7.2, washed in PBS, and then incubated with polyclonal rabbit anti-LIMK1 antibodies, anti-Ser3(P)cofilin antibodies, and/or Alexa 488-conjugated phalloidin in blocking buffer consisting of PBS with 5% goat serum and 0.2% saponin (Sigma). Primary antibodies were detected with Alexa488 or Alexa568-conjugated goat-anti mouse or rabbit antibodies (Invitrogen). Images were acquired with a Zeiss LSM510 confocal microscope equipped with a C-Apochromat X63, 1.2 oil immersion objective. Confocal sections (1.0-1.5 μm) were collected and saved as 512 × 512-pixel images at 8-bit resolution before import into Adobe Photoshop for compilation. The same instrument was used for FRAP experiments. Here Ra2 cells contained on thin glass-bottomed 8-chamber slides were allowed to attach for 60 minutes in medium before incubation in HEPES-buffered salt solution at 37°C in atmospheric air for FRAP analysis. A circular ROI of 43 × 43 pixels (diameter 6 μm) was chosen corresponding to either plasma membrane or podosomal F-actin. After obtaining 2 baseline images bleaching of β-actin-YFP was performed with 60 iterations at full power of the krypton/argon laser (514 nm line), and subsequently images (depth 0.8 μm) were collected at 5 second intervals for 2-3 minutes at low laser emission. The fluorescence intensity in the photobleached region was normalized to the fluorescence intensity measured in a non-bleached region at the same post-bleaching time point to correct for bleaching during time lapse (usually less than 1-2%). Data were fitted to a fluorescence recovery curve with formula F(t) = (F_0_+(F_inf _* t/t_1/2_))/(1 + (t/t_1/2)_)), where F_0 _is the fluorescence intensity immediately after bleaching and F_inf _is the intensity at infinite times of recovery, to obtain the half-time of recovery t_1/2 _[26].

### F/G- actin ratio measurements

Ra2 cells were collected in PBS without Ca^2+ ^and Mg^2+ ^and then seeded in full growth medium in tissue culture dishes for one hour at 37°C. Subsequently, cells were washed once in ice-cold PBS before lysis with actin stabilization buffer (0.1 M PIPES, pH 6.9, 30% glycerol, 5% DMSO, 1 mM MgSO_4_, 1 mM EGTA, 1% TX-100, 1 mM ATP, and protease inhibitor) on ice for 10 minutes. Cells were dislodged by scraping and the entire extract centrifuged at 4°C for 75 minutes in a tabletop centrifuge at 16.000 g. The supernatant containing G-actin was recovered, and the pellet containing F-actin was solubilised with actin depolymerization buffer (0.1 M PIPES, pH 6.9, 1 mM MgSO_4_, 10 mM CaCl_2_, and 5 μM cytochalasin D). Aliquots of supernatant and pellet fractions were separated on 12% SDS-PAGE gels and then western blotted with monoclonal anti-β-actin antibody. Signal was detected by ECL in a digital dark room and integrated optical band density was used to estimate the cellular F/G-actin ratio.

## Results

### F-actin depolymerizing or stabilizing toxins affect superoxide production in Ra2 microglia

**Figure 1:**
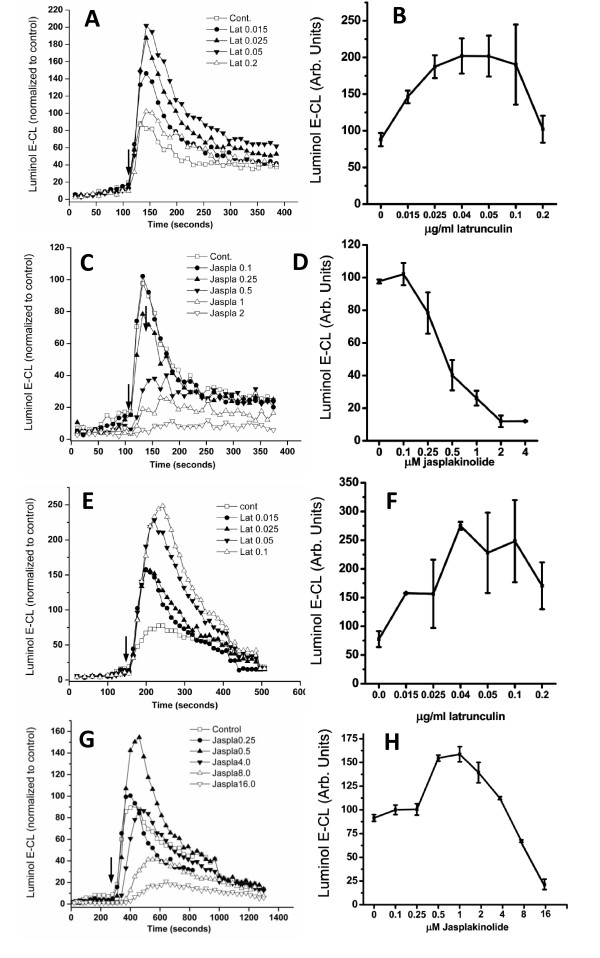
**Latrunculin and jasplakinolide modulate superoxide production in Ra2 microglia**. Superoxide production in Ra2 cells pre-incubated with latrunculin or jasplakinolide as indicated before stimulation with A-D) FMLP or E-H) PMA. The traces presented in the left column show superoxide production in one individual well of an experiment, while the graph on the right show mean ± SEM of peak superoxide production from three independent experiments all in at least three separate runs with duplicate wells on the plate reader. The ordinates show superoxide production as measured by luminol E-CL normalized to control cells not receiving toxin (arbitrary units). Arrows indicate time point of PMA or FMLP injection.

Latrunculin causes F-actin depolymerization by sequestration of monomeric G-actin, and is known to increase the respiratory burst in neutrophils [8,27]. In Figure 1 we compared the effect of latrunculin with that of jasplakinolide, an actin filament-stabilizing toxin, on the FMLP and PMA-induced superoxide production of the murine microglia cell line Ra2 [22], which express all the subunits of the phagocyte NADPH oxidase [28]. We used FMLP because it is a physiological and commonly used phagocyte stimulant. However, any perturbation of the actin cytoskeleton may affect FMLP receptor signaling in addition to effects on the NADPH oxidase complex, for which reason we have also included stimulation with the strong agonist PMA, which works solely on a post-receptor level by PKC activation. Both single well traces (Figure 1A,C,E, and 1G) and mean peak chemiluminescence of at least three independent experiments (Figure 1B,C,F, and 1H) are shown. Latrunculin at a concentration of 40-50 ng/ml induced a 2-3 fold increase in superoxide release measured by luminol-enhanced chemiluminescence (luminol E-CL) for both FMLP and PMA-stimulated cells (Figure 1A,B,E, and 1F). Conversely, jasplakinolide dose-dependently blocked the FMLP-induced response (ca. 90% inhibition at 2 μM), while jasplakinolide in PMA-stimulated cells exerted an enhancing effect on superoxide production (maximum of 60% increase at 1 μM), in the concentration range from ca 0.25-4 μM before becoming inhibitory at concentrations above 4 μM.

### Over-expression of p47phox abolishes the positive and negative effects of actin-perturbing toxins

**Figure 2:**
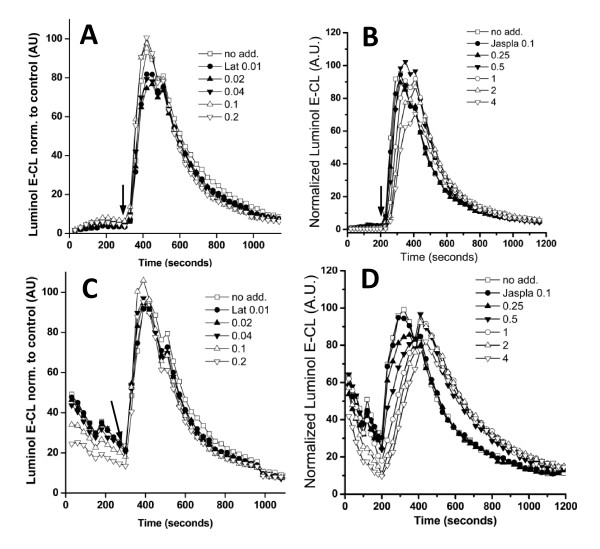
**Over-expression of p47phox alleviates the effects of latrunculin and jasplakinolide**. Superoxide production of Ra2 cells over-expressing p47phox wild type (A,B) or the p47phox (S303D/S304D/S328D) mutant (C,D) and treated with increasing doses of latrunculin (A,C) or jasplakinolide (B,D) before stimulation with PMA. The ordinate in arbitrary units shows luminol E-CL signal normalized to cells receiving no toxin (no add.), and results are presented as mean of at least three independent experiments. Arrows indicate time point of agonist injection.

It has been proposed that cytosolic phox proteins have to be released from an actin-sequestered state for NADPH oxidase assembly [8,16]. We therefore tested if the effects of latrunculin and jasplakinolide on NADPH oxidase activation could be overruled by over-expression of p47phox through titration of actin-related binding sites. Ra2 cells over-expressing p47phox produce five-fold more superoxide than control cells (not shown in Figure 2; see [25]). Figure 2A and 2C shows that latrunculin at concentrations, which caused PMA-stimulated Ra2 cells to produce ca. two-fold more superoxide, only had a modest enhancing effect in Ra2 cells over-expressing wild type p47phox. No further enhancement could be gained by expressing the p47phox(S303D/S304D/S320D) mutant, which mimics phosphorylation and partial activation of p47phox, and is known to induce phosphoinositide and p22phox binding [29], and a basal superoxide production in un-stimulated Ra2 cells [25]. The p47phox over-expressing cells were also partially insensitive to the effects of jasplakinolide (Figure 2B and 2D) although concentrations of more than 0.5 μM caused a delayed peak response of superoxide production.

### Over-expression of LIMK1 in Ra2 microglia regulates cofilin-phosphorylation and the actin cytoskeleton

**Figure 3:**
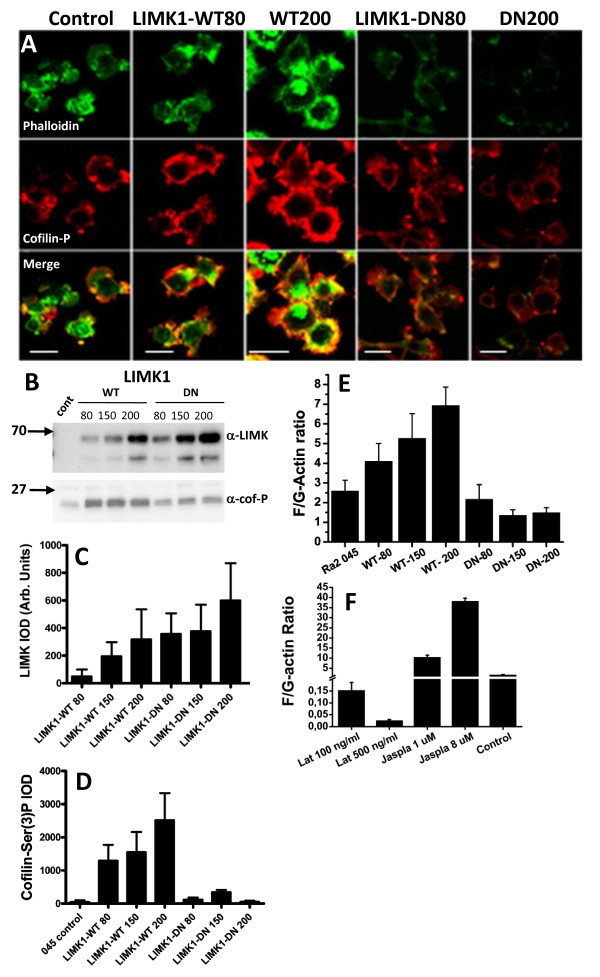
**Over-expression of wild type or dominant negative LIMK1 regulates cofilin phosphorylation and actin polymerization in Ra2 cells**. Ra2 cells received increasing doses (80, 150, or 200 equivalents) of lentivector expressing LIMK1-WT or LIMK1-DN. A) Immunofluorescence of doxycycline-induced Ra2 cell populations using Alexa488-conjugated phalloidin (green) and anti-cofilin-ser3(P) rabbit antibodies (red). Detection gain was adjusted to include the signal for phalloidin or cofilin-ser3(P) from all Ra2 cell populations within the dynamic range and was then kept constant throughout. Bars, 10 μm. B) Expression of LIMK1 protein and effect on phosphorylation of cofilin was determined by western blotting with polyclonal rabbit anti-LIMK1 antibodies and anti-Ser3(P)-cofilin antibodies. Ra2 045 control cells express only the tetracycline-responsive rtTA-transactivator protein. Note that the LIMK1 antibody used only recognizes transgene human LIMK1 protein. C and D) Mean and SEM integrated optical density of western blot bands of LIMK protein (C) and Ser3(P)-cofilin (D) of three individual experiments performed as in B. E and F) The F/G-actin ratios of LIMK1-expressing cells (E) or toxin-treated control cells (F) represent mean and SEM of at least three independent experiments.

We next analyzed the effects of over-expression of wild type and a dominant negative mutant of LIM kinase-1 (LIMK1) as an alternative means of modulating the actin cytoskeleton. Activated LIMK1 shapes the actin cytoskeleton by phosphorylating and thereby inactivating cofilin, which in its active form severs and depolymerizes actin filaments. We created Ra2 cell lines conditionally expressing wild type LIMK1 (LIMK1-WT) or the kinase-dead LIMK1-D406A mutant (LIMK1-DN) at different expression levels (80, 150, and 200 virus dose equivalents). Expression of LIMK1-WT dose-dependently increased phosphorylation of serine-3 in cofilin as expected (Figure 3A, B and 3C) and increased F/G-actin ratios in Ra2 cells (Figure 3E). Curiously, while LIMK1-DN decreased F/G-actin ratios, at all levels of expression it slightly increased the levels of phosphorylated cofilin above the levels of control cells (Figure 3B). We show in Additional File 1 that in fact introducing dominant negative mutants of any of the proteins in the important VAV1, Rac1, PAK1, LIMK1 signaling axis slightly increases p-cofilin levels above control cells, possibly because altered PAK1 or RhoA activation (which is negatively regulated by Rac1) changes the tonus of cofilin phosphatases. For comparison F/G-actin ratios in Ra2 045 control cells treated with select concentrations of latrunculin and jasplakinolide are shown in Figure 3F.

### Wild type and dominant negative LIMK1 modulate superoxide production in Ra2 cells

**Figure 4:**
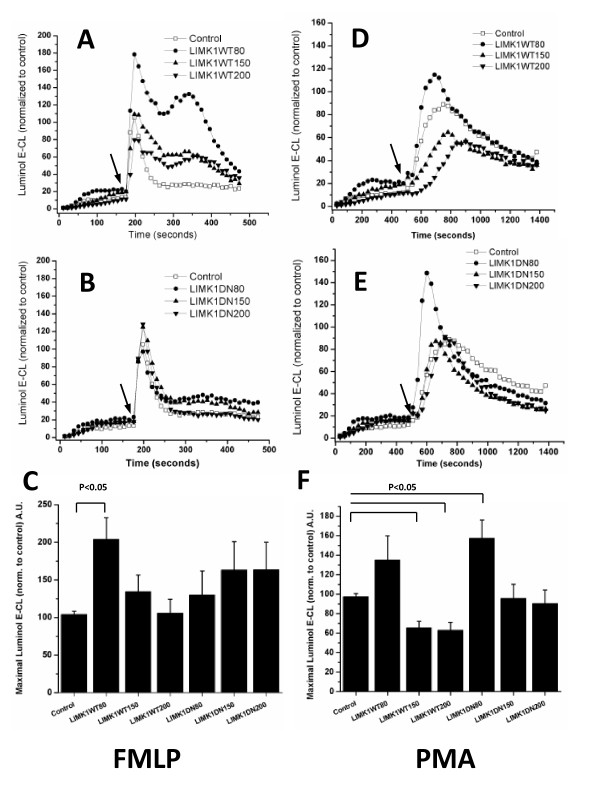
**Effects of LIMK1-WT or LIMK1-DN expression on NADPH oxidase activity**. Ra2 cells expressing LIMK1-WT (A,D) or LIMK1-DN (B,E) at increasing protein levels were stimulated with either FMLP (A-C) or PMA (D-F) and superoxide production measured by Luminol E-CL. The graphs represent traces from individual wells of one experiment, where the ordinate shows luminol E-CL normalized to control cells in arbitrary units; arrows indicate time point of agonist injection. The bar graphs shows peak luminol E-CL response for FMLP (C) or PMA (F) stimulated superoxide production, and represents mean and SD of at least three independent experiments. Differences statistically significant at the P < 0.05 (one sample t-test) level are indicated.

Having confirmed that LIMK1 expression regulates cofilin and F-actin in Ra2 cells, we then examined the effect on FMLP or PMA-elicited superoxide production. Expression of LIMK1-DN dose-dependently increased the FMLP-induced respiratory burst but only expression of LIMK1-WT protein at low levels (Ra2 LIMK1-WT80 cells) significantly induced a two-fold increase in superoxide release (P < 0.05, one sample t-test; Figure 4A and 4C). After PMA stimulation, Ra2 LIMK1-DN80 cells had a significantly (P < 0.05) increased NADPH oxidase activity compared to control cells, and again expression of low levels of LIMK1-WT (WT80) caused an increased superoxide production, while higher levels in WT150 and WT200 Ra2 cells significantly (P < 0.05) decreased the superoxide output with up to 40% (Figure 4D-F). Therefore, despite opposing effects of transgene on F/G-actin ratio both LIMK1-WT80 and LIMK1-DN80 Ra2 cells increased superoxide production up to two-fold following FMLP or PMA stimulation. PAK1 increases FMLP and PMA-stimulated superoxide generation in Ra2 microglia through phosphorylation (partial activation) of the cytosolic p47phox subunit [25], but we also considered that PAK1 through activation of LIMK1 [30] might additionally contribute to NADPH oxidase activity by modulating actin dynamics. However, analysis of superoxide production in a series of Ra2 cell lines co-expressing PAK1 and LIMK1 mutants indicates a parallel rather than sequential organization of these two kinases with respect to NADPH oxidase activity (see Additional File 2).

### Cofilin knock-down inhibits the FMLP and PMA-induced respiratory burst

**Figure 5:**
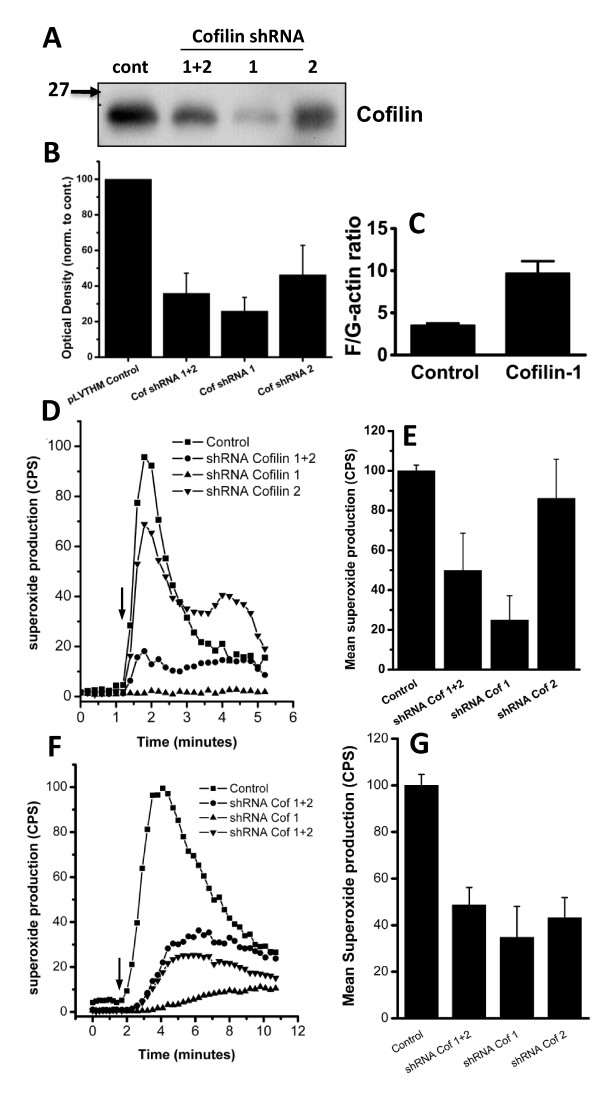
**Cofilin knock-down increases F/G-actin ratio and inhibits superoxide production**. A and B) Levels of cofilin protein after tetracycline induction of two different cofilin shRNAs alone or in combination was determined by western blotting. Control cells refer to Ra2 cells transduced with empty pLVTHM dsRed vector. The bar graphs in B shows mean optical density and SEM of western blot bands derived from three independent experiments. C) Ratio of F- to G-actin in control and cofilin-1 shRNA-expressing Ra2 cells. Data represent mean ± SEM of two independent experiments, each performed in triplicate. D-G) Superoxide production in control or cofilin shRNA-transduced Ra2 cells before and after stimulation with FMLP (D,E) or PMA (F,G) was measured by luminol E-CL. The ordinate of the trace graphs show luminol E-CL normalized to control cells in arbitrary units. Injection of agonist is indicated by arrow. Bar graphs E and G) show normalized luminol E-CL mean and SEM of three independent experiments as performed in A or C, respectively.

We also targeted cofilin directly using shRNA knock-down as described in materials and methods, which decreased the level of cofilin protein in Ra2 cells expressing cofilin-shRNA-1 and shRNA-2 with 75% and 55% relative to control (pLVTHM vector) transduced cells, respectively (Figure 5A and 5B). A knock-down of cofilin would be expected to increase actin polymerization, which was confirmed (Figure 5C). The F/G-actin ratio achieved in Ra2 cofilin-shRNA-1 cells was even higher than that of LIMK1-WT200 cells (10 ± 1 and 6.9 ± 0.9, respectively). The degree of knockdown of cofilin obtained with either cofilin shRNA correlated with a similarly decreased superoxide production (Figure 5, D-G). Cofilin shRNA-1 reduced the respiratory burst following FMLP stimulation with roughly 80% and following PMA stimulation with 70%.

### FRAP analysis of actin recovery rates in Ra2 cells expressing β-actin-YFP

**Figure 6:**
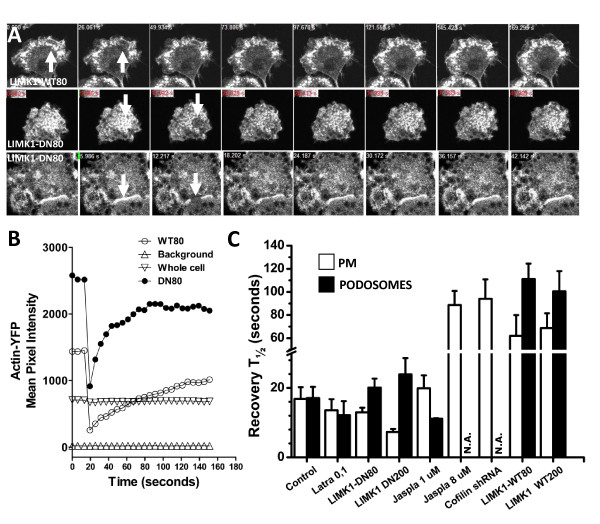
**FRAP experiments reveal widely different actin recovery half-times in transduced Ra2 cells**. Ra2 cell populations as indicated were superinfected with lentivectors expressing β-actin-YFP and used for FRAP. A) Panels (top, LIMK1-WT80 cells; two lower panels, LIMK1-DN80 cells) show time-lapse series of live Ra2 cells where the ROI bleached by the laser beam is indicated by arrows (the frame before and after bleach). In the top and middle row (LIMK1-WT80 and LIMK1-DN80 cells, respectively) the ROI covers podosomal structures, while the lower panel (LIMK1-DN80 cells) shows a ROI covering the opposing plasma membranes of two cells. B) Mean pixel intensity of β-actin-YFP fluorescence is depicted over time before and after bleach (bleach is at time point three) for LIMK1-WT80 cells, together with whole cell and background intensity (white symbols), and for LIMK1-DN80 cells (black). C) Mean pixel intensity of β-actin-YFP fluorescence was fitted to a curve for FRAP recovery to obtain the mean half-time of recovery for either plasma membrane or podosome ROI's for the indicated cell lines or drug-treated cells. The ordinate shows the recovery time in seconds and mean and SEM is based on FRAP curves obtained from at least ten different cell profiles.

F/G-actin ratios derived by end point measurements of F- and G-actin says little or nothing about the dynamics of the actin cytoskeleton, and we therefore considered that turn-over rates could be a better correlate of NADPH oxidase activity than F/G- actin ratios (compare Figure 3F with 4C, F; Figure 3G with 1B, D, F, H). We therefore performed FRAP analysis of β-actin-YFP-expressing Ra2 cells to obtain the actin recovery half time (recovery t_1/2_), which is a measure of actin off- and on-rates combined (Figure 6). We chose two different types of regions of interest (ROI) to work with: podosomal F-actin and cortical, plasma membrane-associated F-actin (see representative examples in Figure 6A and Additional Files 7, 8, 9). FRAP measurements were performed on Ra2 045 control cells, Ra2 LIMK1-WT80/200 and -DN80/200 cells, Ra2 cofilin shRNA-1 cells, and Ra2 cells treated with concentrations of latrunculin and jasplakinolide resulting in maximal positive or negative effect on superoxide production as determined previously (see Figure 1). As seen in Figure 6C, recovery t_1/2 _of actin-YFP fluorescence was depressed slightly by latrunculin or expression of LIMK1-DN at low levels (LIMK1-DN80 cells), which both increase the respiratory burst. Conversely, jasplakinolide at 1 μM, which increases the PMA-stimulated superoxide release to 150% of control levels, caused a modest increase in recovery t_1/2 _in plasma membrane ROI's, while a higher concentration of 8 μM (50% reduction in superoxide release) increased recovery t_1/2 _more than fourfold relative to controls. Expression of LIMK1-WT in WT80 cells distinguished itself from other conditions of enhanced superoxide production (latrunculin 100 ng/ml, LIMK1-DN80, and 1 μM jasplakinolide) by a greatly increased half-time of recovery (62 ± 18 seconds and < 20 seconds, respectively). Of note, cofilin shRNA-1 expression and jasplakinolide treatment both antagonized the formation of podosomes and caused distribution of F-actin to the dorsal plasma membrane.

### Toxins, LIMK1, and cofilin-shRNA result in different in subcellular distribution of F-actin in Ra2 microglia

**Figure 7:**
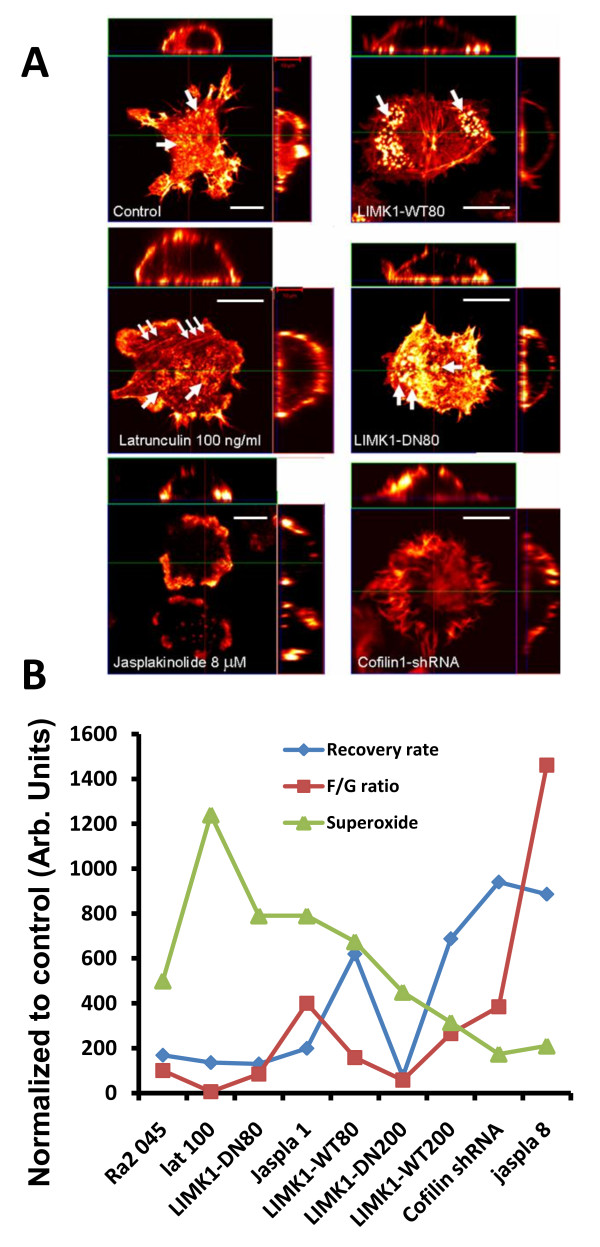
**Changes in morphology and relative changes in superoxide production, F/G-actin ratio, and actin recovery half-time following different experimental perturbations of the actin cytoskeleton**. A) Ra2 LIMK1-WT80, Ra2 LIMK1-DN80, Ra2 cofilin-shRNA1, and Ra2 wild type cells treated for five minutes with 100 ng/ml latrunculin or 8 μM jasplakinolide were paraformaldehyde fixed and stained with Alexa 568-conjugated phalloidin, and then Z-stacks (20-40 planes, ca. 0.25-0.75 μm section width) were acquired. In the case of 8 μM jasplakinolide treatment z-stacks were generated using β-actin-YFP fluorescence as jasplakinolide inhibits phalloidin binding to F-actin. Laser intensity and gain was adjusted to show detail and therefore does not reflect the levels of polymerized actin between cell lines. The images shown represent a XY-view of the most ventral z-plane of the cell (center) while a XZ projection is shown at the top, and a ZY projection on the side. B) For each parameter (superoxide production, F/G-actin ratio, and actin recovery half-time) absolute values derived from experiments above with PMA-stimulated cells were normalized to that of Ra2 045 control cells and depicted on the Y-axis in arbitrary units.

Observations made during FRAP trials prompted us to perform detailed analysis of F-actin distribution by Z-sectioning using fluorophore-conjugated phalloidin to visualize F-actin. Figure 7A shows for each condition a main panel with a XY-view of the most ventral plane of collected Z-stacks, and on the sides of this panel is shown XZ (top) or YZ (side) projections of the reconstructed Z-stack. This analysis confirmed that LIMK1-WT strongly induces podosome-like structures (see movies in Additional Files 3 and 4) and that 8 μM jasplakinolide and cofilin shRNA-1, which most effectively inhibited NADPH oxidase activity, both caused a marked redistribution of F-actin associated with the ventral aspect of the cell membrane to lateral and dorsal membrane (see Additional Files 5 and 6). To allow easier comparison of the effects of different perturbations of the actin cytoskeleton on superoxide production, Figure 7B depicts the normalized values for F/G-actin ratios, actin recovery half-times and superoxide production derived from PMA-stimulated cells overlaid in the same graph (absolute values derived from experiments shown above).

## Discussion

Studies that have addressed the role of the actin cytoskeleton in NADPH oxidase function in phagocytes generally report an increased NADPH oxidase activity following actin depolymerization by latrunculin or cytochalasins, while F-actin stabilization with jasplakinolide decreases superoxide production [8,27,31]. Toxin effects in non-phagocyte cells are varied, possibly due to the 'preassembled' state of NADPH oxidase in some mesenchymal cell types e.g. endothelial cells [32], and actin depolymerization has been noted to enhance [19] or inhibit [18] superoxide production. We show here using conditional over-expression or knock-down of actin-regulatory proteins to modulate the actin cytoskeleton that both actin depolymerization *and *polymerization can enhance stimulated phagocyte NADPH oxidase activity, and further, that neither F/G-actin ratios nor actin recovery rates in the presumed physiological range are directly correlated with NADPH oxidase-mediated superoxide production.

### At what steps of NADPH oxidase assembly and activity is the actin cytoskeleton involved?

In resting cells p40phox, p47phox, and p67phox are thought to be associated in a cytosolic complex [33-35]. In neutrophils [16] and in COS cells [8] binding of p40phox to moesin exerts a restraining effect on phox protein translocation and NADPH oxidase activity. This suggests that cytosolic phox proteins may be tied to F-actin at rest, and is in line with our observation that over-expression of p47phox is sufficient to overcome effects of latrunculin or jasplakinolide on NADPH oxidase activity, likely by increasing the soluble pool of activated phox proteins available to cyt b_558_. A constant exchange of cyt b_558_-associated Rac1/2 and cytosolic phox proteins with new counterparts from cytosol is required to sustain NADPH oxidase activity [10,11].

The autonomy of the translocation of cytosolic phox proteins to the membrane is such that redistribution of p40/p47/p67phox proteins does not necessarily require either cyt b_558 _[12] or inositol lipids [4,36,37], which are otherwise powerful determinants of membrane recruitment of p47phox or p40phox expressed alone [3,7]. At least in mesenchymal cell types cortactin and moesin are important for guiding cytosolic phox proteins to the membrane [15,18,36]. Depending on the relative binding affinities of cytosolic phox proteins and actin-associated proteins for membrane attractors, cytosolic phox proteins may be facilitated in membrane translocation by 'piggy-backing' on coronin, moesin, or cortactin, which bind p40phox and p47phox and are themselves recruited to the plasma membrane following cell activation, incidentally, by intracellular mediators that also participate in NADPH oxidase assembly [16,38,39]. It is not in general known how phosphorylation of p40phox and p47phox relates to binding of actin-associated proteins, but notably, phosphorylated p47phox binds TRAF4 with increased affinity [40]. TRAF4 in turn binds the focal adhesion scaffold protein Hic5 [13]. At the membrane F-actin, actin-regulatory proteins, inositol lipids, and cyt b_558 _present binding sites for newly recruited phox proteins, but it is currently unknown if there is a sequence to binding. However, the failure of p47phox to translocate to the membrane in the absence of WAVE1 [15] or moesin [36], and the premature loss of p47phox, concurrent with the normal loss of F-actin and coronin (within minutes), from newly formed phagosomes in CGD neutrophils devoid of cyt b_558 _[12], suggest that actin-binding proteins may serve as the initial docking station for cytosolic phox proteins before contacts with cyt b_558 _are established. Once the NADPH oxidase holoenzyme is assembled and superoxide production commences, at least in the reconstituted, cell-free system, ongoing actin polymerization increases duration of NADPH oxidase activity [41]. Additionally, in macrophage-like U937 cells cofilin inactivation through LIMK1 activity or anti-sense RNA increases superoxide production following phagocytosis [42-44]. These studies may agree with the positive effect on superoxide production of actin polymerization induced by 1 μM jasplakinolide or LIMK1-WT at low expression levels in Ra2 microglia (158 ± 8% and 135 ± 25% of control values, respectively) as well as the prolonged response of FMLP-stimulated Ra2 cells expressing LIMK1-WT (Figure 4A). However, when we specifically examined if PAK1 could contribute to NADPH oxidase activity by LIMK1-directed actin reorganization, we found no evidence of such a role for PAK1 and LIMK1 (see Additional File 2). It is not known if NADPH oxidase disassembly relates to the state of the immediately surrounding actin cytoskeleton, but in the reconstituted cell-free system actin depolymerization decreases NADPH oxidase activity [41].

### NADPH oxidase activity, F/G- actin ratios, and actin recovery half times

As summarized in Figure 7, conditions that resulted in higher than control values of superoxide production were characterized by widely different actin recovery half times (13.0 ± 1.4 to 62.0 ± 18) and F/G-actin ratios (0.15 ± 0.03 to 10.4 ± 1.0). However, at critical thresholds defined in Ra2 microglia by the transition from LIMK1-WT80 to -WT200 expression levels (WT80/200: recovery rates 62 ± 18/68 ± 12 and F/G-actin ratios 4.1 ± 0.9/6.9 ± 0.9) and in the lower end by LIMK1-DN200 (7.3 ± 0.8/1.5 ± 0.3) superoxide production is repressed to ca. 50% of that of control Ra2 cells (16.8+-3.4/2.6+-0.6) and any further increases/decreases in actin recovery rate progressively abolishes superoxide production. The most effective block of superoxide production was under conditions of enforced actin polymerization by either cofilin knock-down (70-80% inhibition) or high concentrations (> 8 μM) of jasplakinolide (80% inhibition), which yielded actin recovery half times of 94 ± 16.9 and 88.6 ± 12.2 seconds, respectively. These conditions however also uniquely caused the redistribution of F-actin from ventral to lateral and dorsal cell surfaces, which could maybe interfere with NADPH oxidase function by disturbing any substratum directed signaling required for oxidase assembly.

**Figure 8:**
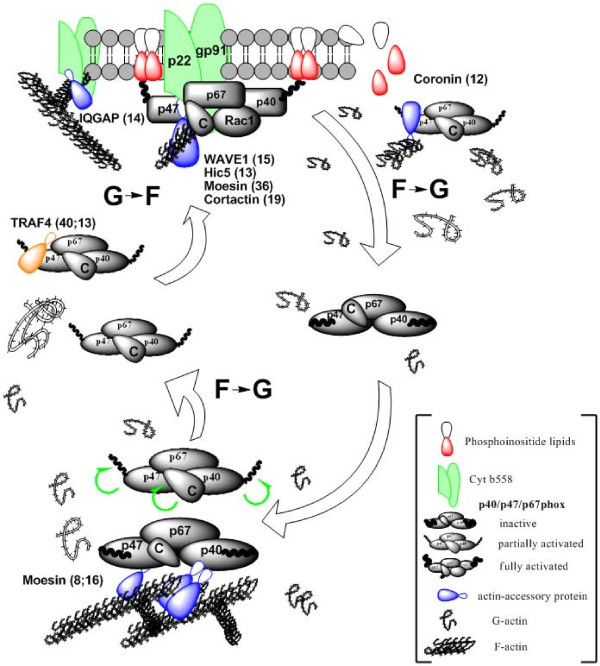
**Model of p40/p47/p67phox membrane translocation and potential roles for the actin cytoskeleton and actin-regulatory proteins in NADPH oxidase assembly and function**. At rest the p40/p47/p67phox complex is tethered to F-actin either directly or indirectly through for example moesin (bottom). Following cell activation p40phox and p47phox are activated by phosphorylation to acquire phosphoinositide and cyt b_558_-binding properties (green curved arrows) and loss of tight binding to F-actin. In addition, phosphorylation may lead to binding of actin-associated proteins or adaptors like TRAF4 to the phox protein complex. This membrane recruitment part of the cycle is supported by actin depolymerization (F- to G-actin conversion). At the membrane p47phox bind cortical actin, phosphoinositides in the membrane and cyt b_558 _to position p67phox for catalysis of electron transfer to cyt b_558_. The specific role of actin-associated proteins in the recruitment, retention, or dissociation of p40/p47/p67phox at the membrane is unclear but WAVE1, moesin, and cortactin are likely candidates as recruiter proteins. Once the NADPH oxidase holo-enzyme has been assembled the magnitude and duration of NADPH oxidase activity is augmented by polymerization of actin (G- to F-actin conversion). Note, that cyt b_558 _itself is tightly associated with cortical actin and can be redistributed through the action of IQGAP. Evidence for the role of actin in disassembly of NADPH oxidase and deactivation of p40/p47/p67phox is unavailable, but likely p40/p47/p67phox returns to a tethered state with F-actin as end-point following dephosphorylation of p47phox.

Therefore we conclude that at least in microglia actin polymerization and depolymerization can both enhance NADPH oxidase activity. This apparently contradictory result is probably reconciled by the cyclic nature of NADPH oxidase assembly during superoxide production: cytosolic phox proteins and Rac1/2 bound to cyt b_558 _are constantly exchanged with new subunits [10,11], which require release from F-actin (mobilization), translocation to membrane (actin or actin-associated protein facilitated), and tethering to membrane (initial contacts with cortical F-actin or associated proteins?) as illustrated in Figure 8. During cell activation these processes may be aided preferentially by induced F-actin depolymerization. Superimposed on this subunit cycling is the mechanic influence of F-actin on the assembled and functional NADPH oxidase whose activity is increased and prolonged by active actin polymerization [41]. Therefore, increasing the concentration of an actin polymerizing or depolymerizing factor may increase NADPH oxidase activity, but only to a point, where after one or more steps in the continuous assembly/disassembly cycle of cytosolic phox proteins with cyt b_558_, facilitated by the opposite actin fate, will be inhibited to a degree where it negatively affects superoxide release. Our results also imply that NADPH oxidase function in stimulated phagocytes always will be in an enhanced state of activity regardless of the very different alterations of the actin cytoskeleton required to effect different immune cell tasks such as chemotaxis, invasion, phagocytosis, redox signaling, or immunological synapse formation. Such a capacity of the respiratory burst to accommodate the plasticity of the actin cytoskeleton may be particularly relevant for microglia, which undergoe dramatic changes in morphology following cell activation going from a highly elongated and ramified morphology to a rounded, amoeboid shape.

## Abbreviations

FMLP: N-Formylmethionine leucyl-phenylalanine; FRAP: fluorescence recovery after photobleaching; LIMK1: LIM Kinase-1; Luminol E-CL: luminol-enhanced chemiluminescence; PAK1: p21-activated Kinase-1; PBS: phosphate-buffered saline; PMA: Phorbol myristate acetate; TRAF4: TNF receptor-associated factor 4; WAVE1: Verprolin homology domain-containing protein 1.

## Supplementary Material

Additional file 1 **The effects of dominant negative mutants of VAV1, Rac1, and LIMK1 on cofilin phosphorylation**. Cell extracts of Ra2 cells conditionally expressing VAV1-L213A, Rac1-N17, or LIMK1-WT or -DN protein were separated on SDS-PAGE gels at 10 ug/lane, transferred to PVDF membranes, and western blotted with anti-Ser(3)P-cofilin antibodies. Click here for file

Additional file 2 **NADPH oxidase activity-modulating PAK1 and LIMK1 mutants additively enhance or repress superoxide production**. We have recently shown that PAK1 increases FMLP and PMA-stimulated superoxide generation in Ra2 microglia through phosphorylation (partial activation) of the cytosolic p47phox subunit [25]. In light of the observations made in Figure 1 and 4 we considered that PAK1 through activation of LIMK1 might additionally contribute to NADPH oxidase activity by modulating actin dynamics. We therefore co-expressed dominant positive PAK1-T423E or dominant negative PAK1-K299A with LIMK1 in Ra2 cells to analyze the sequential or parallel organization of PAK1 and LIMK1 with respect to NADPH oxidase activation. LIMK1 transgene was expressed in comparable levels in the doubly transduced cell populations (at the level of LIMK1-WT80 and LIMK1-DN80 cells) and regulated the levels of phosphorylated cofilin (File 2A) and actin F/G-ratio (File 2B). Additional File 2C and 2D show peak superoxide production as measured with luminol E-CL following stimulation with FMLP or PMA. Note that co-expression of PAK1-T423E and LIMK1-DN additively increased the superoxide production. Conversely, co-expression of PAK1-K299A, which on its own has little effect on the FMLP response, but inhibits the PMA-induced signal with approximately 50% [25], and LIMK1-WT additively decreased superoxide production to ca. 25% of controls in PMA stimulated Ra2 cells. The results therefore indicate that at least in response to non-particulate stimulants FMLP and PMA, PAK1 activity enhances NADPH oxidase superoxide production without need for LIMK1 activity. The effects on NADPH oxidase in Ra2 microglia co-expressing PAK1 and LIMK1. A) Cell extracts of Ra2 cells conditionally expressing PAK1 or LIMK1 protein alone or in combination were western blotted with anti-myc (PAK1), anti-LIMK1, or anti-Ser(3)P-cofilin antibodies, respectively. The western blot is representative of two independent experiments. B) F/G-actin ratio was determined as before. Results represent mean ± SEM of three independent experiments. C and D) Superoxide production in Ra2 cells expressing PAK1 and LIMK1 protein alone or in combination was measured by luminol E-CL in FMLP (D) or PMA (E) simulated cells as before. The ordinate represents mean ± SEM of peak luminol E-CL normalized to control cells derived from at least five independent experiments. Differences statistically significant from control at the P < 0.05 level (one sample t-test) are indicated. Click here for file

Additional file 3 **Morphology of the actin cytoskeleton in Ra2 LIMK1-WT200 cells**. Ra2 LIMK1-WT200 cells were fixed, permeabilized and stained for F-actin using Alexa568-conjugated phalloidin. The movies show 3 D projections of reconstructed confocal z-stacks obtained as 20 confocal sections approximately 0.3-0.5 μm thick for a single cell (a) or a group of cells (b). Click here for file

Additional file 4 **Morphology of the actin cytoskeleton in Ra2 LIMK1-WT200 cells**. Ra2 LIMK1-WT200 cells were fixed, permeabilized and stained for F-actin using Alexa568-conjugated phalloidin. The movies show 3 D projections of reconstructed confocal z-stacks obtained as 20 confocal sections approximately 0.3-0.5 μm thick for a single cell (a) or a group of cells (b). Click here for file

Additional file 5 **Morphology of the actin cytoskeleton in Ra2 shRNA cofilin-1 cells**. Ra2 shRNA cofilin-1 cells were fixed, permeabilized, and stained for F-actin using Alexa568-conjugated phalloidin. The movies show 3 D projections of reconstructed confocal z-stacks obtained as 20 confocal sections approximately 0.3-0.5 μm thick for a single cell (a) or a group of cells (b). Click here for file

Additional file 6 **Morphology of the actin cytoskeleton in Ra2 shRNA cofilin-1 cells**. Ra2 shRNA cofilin-1 cells were fixed, permeabilized, and stained for F-actin using Alexa568-conjugated phalloidin. The movies show 3 D projections of reconstructed confocal z-stacks obtained as 20 confocal sections approximately 0.3-0.5 μm thick for a single cell (a) or a group of cells (b). Click here for file

Additional file 7 **FRAP reveals different actin dynamics**. FRAP experiments performed on Ra2 LIMK1-WT80 cells (a) or Ra2 LIMK1-DN80 cells (b and c). Shown are the types of regions of interest (ROI) used, namely podosomes (a, and b) or plasma membrane (c), here the membranes of two opposing cells. Click here for file

Additional file 8 **FRAP reveals different actin dynamics**. FRAP experiments performed on Ra2 LIMK1-WT80 cells (a) or Ra2 LIMK1-DN80 cells (b and c). Shown are the types of regions of interest (ROI) used, namely podosomes (a, and b) or plasma membrane (c), here the membranes of two opposing cells. Click here for file

Additional file 9 **FRAP reveals different actin dynamics**. FRAP experiments performed on Ra2 LIMK1-WT80 cells (a) or Ra2 LIMK1-DN80 cells (b and c). Shown are the types of regions of interest (ROI) used, namely podosomes (a, and b) or plasma membrane (c), here the membranes of two opposing cells. Click here for file
