# Supplementary figures and images for: Effects of F/G-actin ratio and actin turn-over rate on NADPH oxidase activity in microglia
Source: BMC Immunol. 2010 Sep 8;11:44. doi: 10.1186/1471-2172-11-44 (PMC2944333; doi:10.1186/1471-2172-11-44)

045 Control

Rac1-N17

VAV1-L213A

LIMK WT

LIMK DN

21 kDa

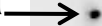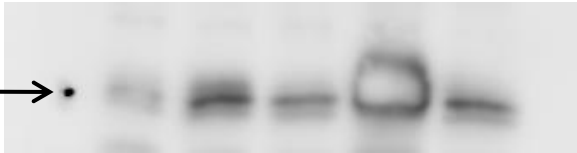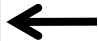

**P-cofilin**

Supplement: Additional file 1 — The effects of dominant negative mutants of VAV1, Rac1, and LIMK1 on cofilin phosphorylation. Cell extracts of Ra2 cells conditionally expressing VAV1-L213A, Rac1-N17, or LIMK1-WT or -DN protein were separated on SDS-PAGE gels at 10 ug/lane, transferred to PVDF membranes, and western blotted with anti-Ser(3)P-cofilin antibodies. [file 1471-2172-11-44-S1.PDF]

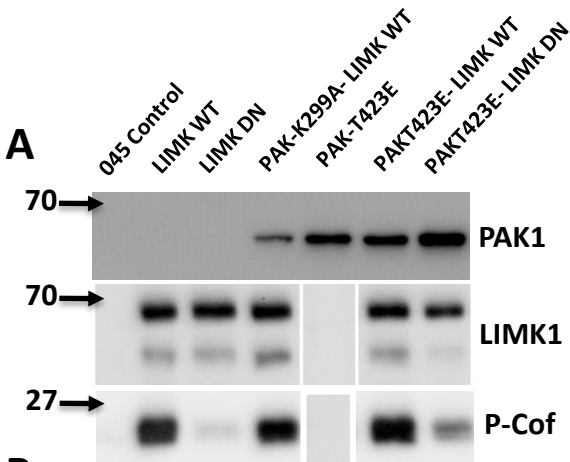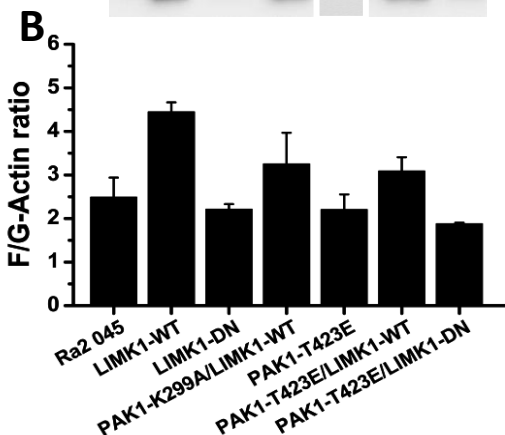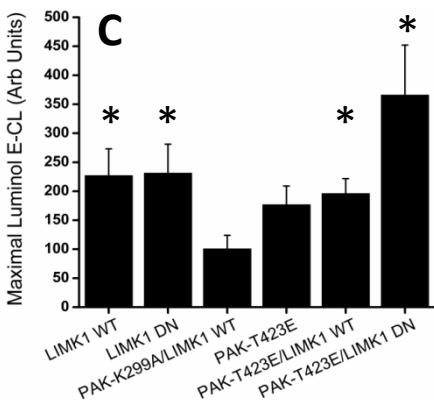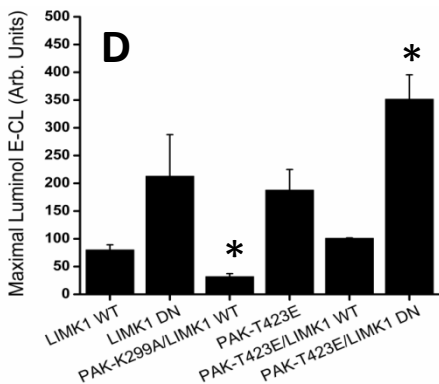

Supplement: Additional file 2 — NADPH oxidase activity-modulating PAK1 and LIMK1 mutants additively enhance or repress superoxide production. We have recently shown that PAK1 increases FMLP and PMA-stimulated superoxide generation in Ra2 microglia through phosphorylation (partial activation) of the cytosolic p47phox subunit [25]. In light of the observations made in Figure 1 and 4 we considered that PAK1 through activation of LIMK1 might additionally contribute to NADPH oxidase activity by modulating actin dynamics. We therefore co-expressed dominant positive PAK1-T423E or dominant negative PAK1-K299A with LIMK1 in Ra2 cells to analyze the sequential or parallel organization of PAK1 and LIMK1 with respect to NADPH oxidase activation. LIMK1 transgene was expressed in comparable levels in the doubly transduced cell populations (at the level of LIMK1-WT80 and LIMK1-DN80 cells) and regulated the levels of phosphorylated cofilin (File 2A) and actin F/G-ratio (File 2B). Additional File 2C and 2D show peak superoxide production as measured with luminol E-CL following stimulation with FMLP or PMA. Note that co-expression of PAK1-T423E and LIMK1-DN additively increased the superoxide production. Conversely, co-expression of PAK1-K299A, which on its own has little effect on the FMLP response, but inhibits the PMA-induced signal with approximately 50% [25], and LIMK1-WT additively decreased superoxide production to ca. 25% of controls in PMA stimulated Ra2 cells. The results therefore indicate that at least in response to non-particulate stimulants FMLP and PMA, PAK1 activity enhances NADPH oxidase superoxide production without need for LIMK1 activity. The effects on NADPH oxidase in Ra2 microglia co-expressing PAK1 and LIMK1. A) Cell extracts of Ra2 cells conditionally expressing PAK1 or LIMK1 protein alone or in combination were western blotted with anti-myc (PAK1), anti-LIMK1, or anti-Ser(3)P-cofilin antibodies, respectively. The western blot is representative of two independent experiments. B) F/G-actin [file 1471-2172-11-44-S2.PDF]
